# Making brain–computer interfaces as reliable as muscles

**DOI:** 10.1088/1741-2552/addd47

**Published:** 2025-07-28

**Authors:** Jonathan R Wolpaw

**Affiliations:** National Center for Adaptive Neurotechnologies, Albany Stratton VA Medical Center and State University of New York at Albany, 113 Holland Ave, Albany, NY 12208, United States of America

**Keywords:** brain–computer interface, brain-machine interface, alternative communication, heksor, negotiated equilibrium, synthetic heksor, motor skills

## Abstract

**Objective.:**

While brain–computer interfaces (BCIs) can restore basic communication to people lacking muscle control, they cannot yet restore actions that require the extremely high reliability of natural (i.e. muscle-based) actions. Most BCI research focuses on neural engineering; it seeks to improve the measurement and analysis of brain signals. But neural engineering alone cannot make BCIs reliable.

**Approach.:**

A BCI does not simply decode brain activity; it enables its user to acquire a skill that is produced not by nerves and muscles but rather by the BCI. Thus, BCI research should focus also on neuroscience; it should seek to develop BCI skills that emulate natural skills.

**Main results.:**

A natural skill is produced by a network of neurons and synapses that may extend from cortex to spinal cord. This network has been given the name *heksor*, from the ancient Greek word *hexis*. A heksor changes through life; it modifies itself as needed to maintain the key features of its skill, the attributes that make the skill satisfactory. Heksors overlap; they share neurons and synapses. Through their concurrent changes, heksors keep neuronal and synaptic properties in a *negotiated equilibrium* that enables each to produce its skill satisfactorily. A BCI-based skill is produced by a *synthetic heksor*, a network of neurons, synapses, and software that produces a BCI-based skill and should change as needed to maintain the skill’s key features.

**Significance.:**

A synthetic heksor shares neurons and synapses with natural heksors. Like natural heksors, it can benefit from multimodal sensory feedback, using signals from multiple brain areas, and maintaining the skill’s key features rather than all its details. A synthetic heksor also needs successful co-adaptation between its central nervous system and software components and successful integration into the negotiated equilibrium that heksors establish and maintain. With due attention to both neural engineering and neuroscience, BCIs could become as reliable as muscles.

## 1. Introduction

Throughout life, the central nervous system (CNS) acquires and maintains a host of useful behaviors, commonly known as skills. These range from the simple flexion withdrawal reflexes acquired in utero and early postnatal life (Granmo et al 2008, Schouenborg 2008), through standard skills such as locomotion and speech, to the most sophisticated athletic and cognitive performances. All these natural skills are produced through muscles. In contrast, brain–computer interfaces (BCIs) enable the CNS to acquire skills that are produced not through muscles but rather through brain signals, such as neuronal action potentials (single-neuron activity) or electroencephalographic (EEG), magnetoencephalographic, electrocorticographic, or stereo-EEG activity (Wolpaw et al 2002, Luo et al 2022, Pandarinath and Bensmaia 2022).

**Figure 1.**
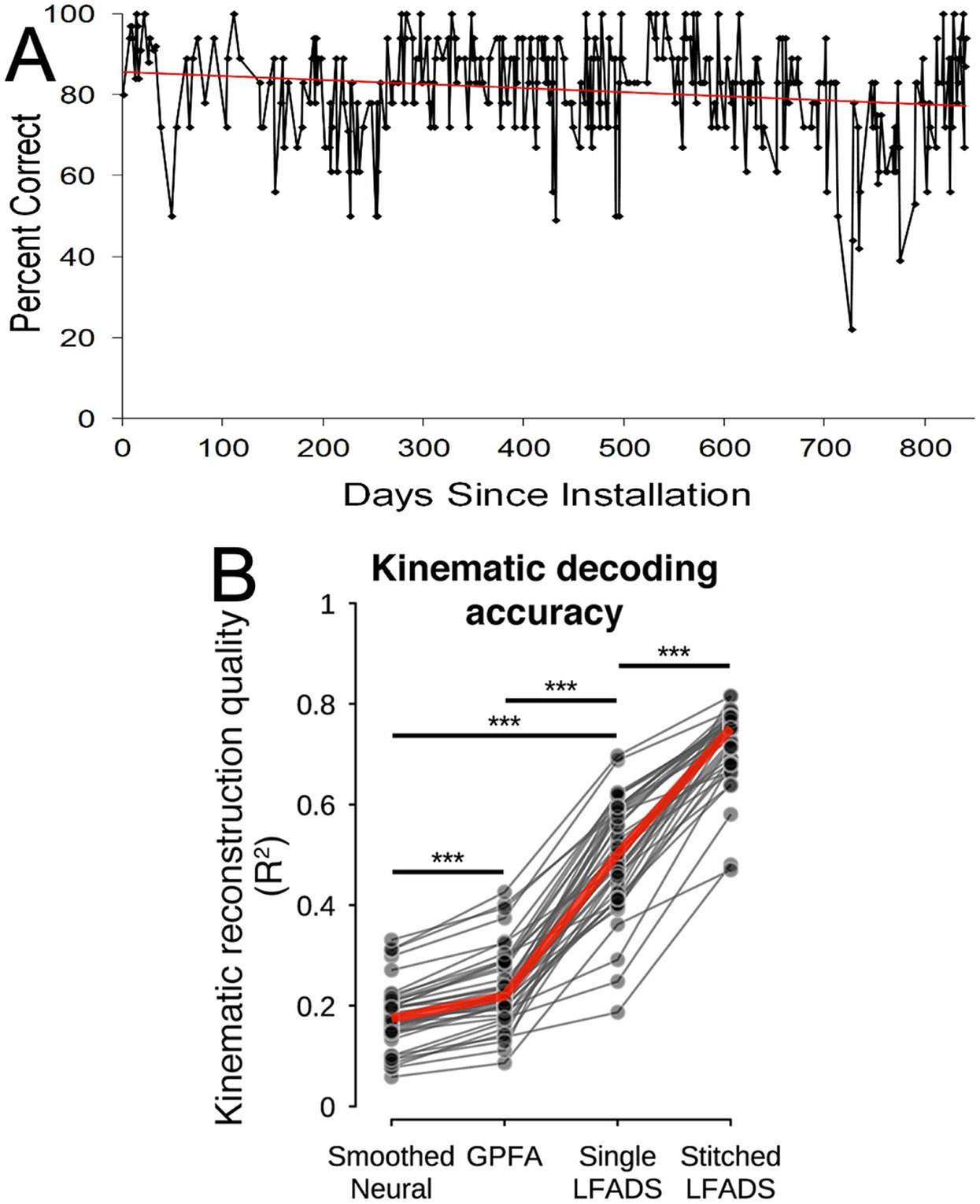
Current BCIs are not reliable. (A) A man with ALS who could no longer use his eye-gaze system used a home-based BCI to interact with family and friends and to supervise his NIH-funded research laboratory. His median performance on periodic copy-spelling tests was 83% (chance accuracy 1.4%) and was stable over this long period (r=−0.07, not significant), but it varied widely from day to day. Thus, effective communication took longer on some days than on others. The high variability was not ascribable to technical issues. (Unpublished data from our study described in Sellers et al 2010). (B) Increasingly sophisticated attention to latent factors and neural dynamics produced progressive increase in mean accuracy (red line) for decoding movement kinematics from motor cortex neuronal activity recorded in multiple sessions over 5 months (gray dots). However, inter-session variation in accuracy remained high, even as mean accuracy greatly improved. Inter-session variation was as high for the most accurate method as it was for the least accurate. (Reproduced from Pandarinath et al 2018 CC BY 4.0.).

Current BCIs are useful for actions that do not need to be completely reliable; actions in which mistakes do not have unacceptable consequences. They can provide basic communication and simple, non-dangerous movement capacities to people who lack effective muscle control such as those with severe cerebral palsy, brainstem stroke, or late-stage amyotrophic lateral sclerosis (ALS) (e.g. Vansteensel et al 2016, Wolpaw et al 2018). For example, our laboratory provided a BCI that restored communication to a man with ALS who could no longer use his eye-gaze system (Sellers et al 2010). For more than two years, he used the BCI to interact with family and friends and to supervise his NIH-funded research lab. Figure 1(A) shows the BCI’s accuracy in copy-spelling tests over this extended period. While accuracy averaged 83% (chance accuracy 1.4%) and was stable over the years, its day-to-day performance remained highly variable, even as the user practiced the skill over months and years. This persistent variability could not be ascribed to technical issues. The high variability meant that successful communication took longer on some days than on others, but it did not have unacceptable consequences. BCIs now in development can provide more complex control (e.g. Willett et al 2021). However, they too lack the reliability that would make them suitable for actions in which errors are not acceptable. In their impressive presentation of the improvement in BCI accuracy provided by better analysis methods, Pandarinath et al (2018) provide a striking illustration of the reliability problem. Figure 1(B) shows that, as average BCI performance increases with improved analysis, the variation in performance remains high. Better signal analysis greatly improves mean accuracy, but it does not reduce the high session-to-session variation in accuracy. Clearly, something more is needed.

This paper considers how BCIs might be made reliable enough for actions in which errors can have unacceptable consequences. For example, how can we produce a BCI that most people would be willing to use to move along the edge of a high cliff? How can BCI performance of such actions become as reliable as muscle-based performance would be?

BCI research and development has focused primarily on neural engineering. Investigators seek new and better ways to measure brain signals. They are developing methods to measure more signals from more locations with more precision, methods to record specific signals reliably from minute to minute, day to day, and ultimately year to year, and methods that are safe and can be seamlessly integrated into a person’s life without undue demands on the person or on others. They also seek new and better ways to analyze brain signals so as to determine a person’s intentions quickly and reliably. This is commonly described as decoding brain activity. Despite these advances, BCI-based skills still remain less reliable than muscle-based skills. Neural engineering alone is not enough.

Because the goal is to make BCI-based skills as good as natural muscle-based skills, research and development needs to focus also on neuroscience, especially on how muscle-based skills are acquired and maintained (Wolpaw and Wolpaw 2012). As described below, a number of research groups are beginning to do this; they are trying to enable BCI-based skills to function more like muscle-based skills. This paper seeks to further encourage and develop this initiative by considering how the acquisition and maintenance of BCI-based skills might best emulate the process by which muscle-based skills are acquired and maintained.

## 2. Muscle-based skills versus BCI-based skills

The research of the past half-century has shown that the CNS remains plastic through life. From cortex to spinal cord, neurons, synapses, and glia change continually to support the many skills that an individual acquires and maintains. With this realization of life-long ubiquitous plasticity has come new understanding of skills. It is now understood that a skill is produced by a network of neurons and synapses that may extend from cortex to spinal cord. The skill is acquired and maintained by ongoing plasticity in neuronal and synaptic properties throughout the network. This network has recently been given the name *heksor* based on an appropriate Greek root. A *heksor* is a network of neurons and synapses that produces a skill and changes (i.e. adapts) continually based on feedback to maintain the key features of the skill—the attributes that make it satisfactory (Wolpaw and Kamesar 2022 for full presentation). For example, the key features of locomotion include attributes such as right-left symmetry, upright posture, adequate balance, and acceptable metabolic cost. Locomotor muscle activity and kinematics may change; key features are maintained. The recognition of heksors as the agents that produce skilled behaviors was presaged by Bernstein’s concept of skilled behaviors as ‘biodynamic structures that live and develop’ (Bernstein 1967).

**Figure 2.**
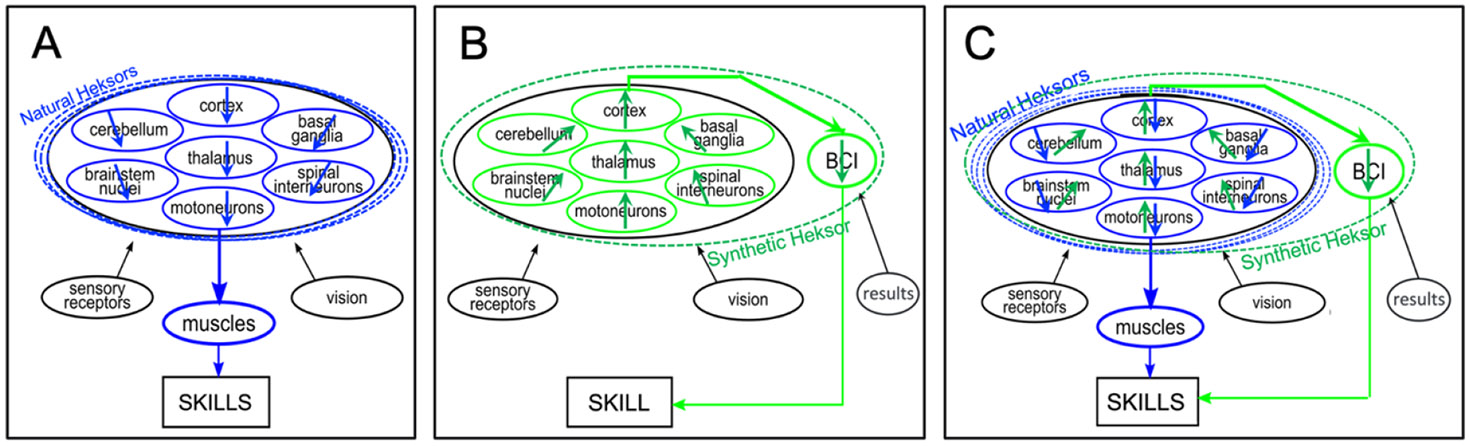
Skill acquisition and maintenance (A) Simple summary of how muscle-based skills are acquired and maintained. It shows the principal regions of the CNS that together produce skill-specific muscle activity, and it indicates the many heksors that share these regions. Each heksor spans multiple regions and shares neurons and synapses with other heksors. The blue arrows represent the ongoing adaptations in each region through which the heksors create a negotiated equilibrium that enables all of them to produce their skills satisfactorily. As the arrow directions illustrate, these adaptations ensure muscle output that is satisfactory for all the heksors. (B) Simple summary of how a BCI-based skill is acquired and maintained. The green arrows represent the ongoing adaptations occurring in both the CNS component and the software component (BCI circle) of the synthetic heksor. As the arrow directions indicate, these adaptations seek to ensure BCI output that is satisfactory for the synthetic heksor. (C) This superimposition of A and B indicates the difficulty of acquiring and maintaining a BCI-based skill. As the direction of the blue arrows shows, all the natural heksors are continually adapting neurons and synapses in each CNS region to control muscles so that each heksor produces its skill satisfactorily. In contrast, as the very different direction of the green arrows shows, the synthetic heksor is continually adapting neurons and synapses in each CNS region, and the BCI software, to control the BCI output so that the synthetic heksor produces its skill satisfactorily. Given the great difference between the goals of their adaptations (indicated by their difference in arrow direction), effective negotiation between the natural heksors and the synthetic heksor is likely to be extremely difficult. In present-day BCIs, the principal loser is the synthetic heksor: BCI performance is not reliable.

Because heksors share neurons and synapses, their concurrent adaptations are a negotiation. Through this negotiation, the heksors keep CNS neurons and synapses in a *negotiated equilibrium* that ensures that each heksor’s skill remains satisfactory. Figure 2(A) is a simple summary of how muscle-based skills are acquired and maintained. It shows the principal regions of the CNS that together produce skill-specific muscle activity, and it indicates the many heksors that share these regions. Each heksor spans multiple regions and shares neurons and synapses with other heksors. The arrows represent the ongoing adaptations in each region through which the heksors create a negotiated equilibrium that enables all of them to maintain the key features of their skills.

Figure 2 shows that BCI-based skills differ in three ways from muscle-based skills. First, and most obviously, they are produced through BCI output, not through spinal motoneuron output. Second, as the upward direction of their green arrows indicates, they are not produced in a top-down fashion in which the ongoing adaptations in each region (blue arrows) seek to optimize the output that controls the muscles. Third, they are not produced by the CNS alone; they are produced by the CNS and the software of the BCI. Thus, a BCI-based skill is produced by what might be called a *synthetic heksor*. A *synthetic heksor* is a network comprised of neurons, synapses, and software. They work together to produce a BCI-based skill; and—if the skill is reliable—they change (i.e. adapt) as needed to maintain the key features of the skill, the attributes that make the skill satisfactory. Like natural heksors, a synthetic heksor may span multiple CNS regions and share neurons and synapses with natural heksors. Figure 2(B) is a simple summary of how a BCI-based skill is acquired and maintained. The arrows represent the ongoing adaptations occurring in both the CNS component and the software component of the synthetic heksor. As the arrow directions indicate, these adaptations seek to produce satisfactory BCI output.

The synthetic heksor concept clarifies the problem of making BCI-based skills as good as muscle-based skills. The challenge is to create synthetic heksors that emulate natural heksors. The neurons, synapses, and software of a synthetic heksor should function together so as to produce BCI skills that are as reliable and rapid as muscle-based skills. To achieve this, a synthetic heksor needs to possess the two essential properties of a natural heksor. First, it needs the ability to adapt to maintain the key features of its skill. Efforts to enhance synthetic heksor adaptation are underway (see below).

Second, a synthetic heksor needs to join effectively in the ongoing negotiation that enables all the heksors—natural and synthetic—to maintain their key features. This is the more difficult problem. Figure 2(C), which overlays figures 2(A) and (B), illustrates why. As the direction of the blue arrows shows, all the natural heksors are continually adapting neurons and synapses in each CNS region to control muscles so that each heksor achieves its key features. In contrast, as the very different direction of the green arrows shows, the synthetic heksor is continually adapting neurons and synapses, and the BCI software, to control BCI output so that the synthetic heksor achieves its key features. This difference in direction means that the ongoing negotiation between the adaptations of the many natural heksors and those of a single synthetic heksor is likely to be an extremely unequal competition in which the synthetic heksor is the inevitable loser. This extreme imbalance likely contributes to the intractable variability seen in BCI performance. Efforts to address this problem have barely begun. While several worthwhile initial steps are described below, a full solution awaits better understanding of how natural heksors negotiate and development of methods that enable synthetic heksors to join in that negotiation.

In sum, BCI research and development has two problems to solve. The first problem is how to optimize the concurrent co-adaptation of the two components of a synthetic heksor—the software component and the neuronal/synaptic component—so that the heksor maintains the key features of its skill. The second problem is how to optimize the concurrent co-adaptation (i.e. negotiation) between the synthetic heksor and the natural heksors with which it shares neurons and synapses. That is, the problem is how to optimize the integration of the synthetic heksor into the negotiated equilibrium that enables all the heksors, natural or synthetic, to maintain the key features of their skills.

## 3. Enhancing co-adaptation within a synthetic heksor

As many studies show (and figure 2(A) illustrates), changes in the neurons and synapses of the natural heksors that produce muscle-based skills are guided by feedback during skill performance and by the final result (Adams 1987, Criscimagna-Hemminger et al 2010, Krakauer and Mazzoni 2011, Roemmich et al 2016, Kim et al 2019, Pacheco et al 2019). These ongoing adaptations reduce errors and improve performance. They shape a natural heksor during its original creation and they enable it to maintain the key features of its skill through life.

For a synthetic heksor, this adaptive process is inherently more complex. It typically entails two adaptive processes: one in the CNS component of the synthetic heksor; and one in the software component (as shown in figure 2(B)). The CNS process is inadequately understood and difficult to control. The software process is fully defined and controllable. The central problem is their interaction; how do they co-adapt to each other so that together they acquire and maintain the key features of the BCI skill produced by the synthetic heksor? Although the need for effective co-adaptation has been recognized for many years (Rosenfeld 1990, Coles and Rugg 1995, Wolpaw et al 2002), only in recent years has it begun to receive substantial conceptual and experimental attention.

Perdikis and Millán (2020) provide a comprehensive review and discussion of BCI co-adaptation. They focus on the difficulty and importance of distinguishing between gains in BCI performance due to user learning (i.e. changes in the CNS component of the synthetic heksor) and gains in performance due to improvements in the decoding of brain activity (i.e. changes in the software component of the heksor). They describe the many uncertainties that remain concerning the nature of BCI user learning and stress the unfortunate paucity of longitudinal closed-loop studies generally, particularly ones that distinguish clearly between user learning and decoder improvement. While these authors have no definitive answers, they conclude that the available evidence suggests that relatively stable BCI decoders (i.e. decoders that adapt slowly or infrequently) are best able to encourage and guide user learning, and thereby improve BCI performance.

Madduri et al (2023) suggest that the co-adaptive interaction between the CNS and software components of a synthetic heksor might be modeled as a game in which the goals of the two players are similar, but not identical. That is, the two components would each seek to achieve the key features that define the BCI-based skill as satisfactory. At the same time, they might differ in how they prioritize key features, or in how they endeavor to achieve them. For example, for a BCI that enables a person to activate the muscles of a limb paralyzed by spinal cord injury (e.g. Wagner et al 2018), the key feature of acceptable metabolic cost could well be more important for the CNS component than for the software component; and the CNS component’s criterion for ‘acceptable’ might decrease as muscle fatigue increased. In another example, in a BCI that translates cortical activity into individual letters to enable a paralyzed person to write (e.g. Willett et al 2021), the CNS component might seek activity concentrated in a relatively small volume of interconnected neurons, while the software component might seek activity that is more broadly distributed across cortex, and thus is more reliably recorded and decoded.

Recent years have also seen productive efforts to enhance co-adaptation in a synthetic heksor. One addresses the need for improved sensory feedback. As noted about, skills, whether muscle-based or BCI-based, are associated with, and dependent on sensory feedback. For a muscle-based skill, this feedback is multimodal, rapid, and ongoing. Proprioceptive, cutaneous, and visual inputs, and often vestibular and auditory inputs as well, provide detailed real-time information about what is happening. This feedback contributes to skill-specific muscle activity, and it also guides the ongoing adaptations through which a natural heksor maintains the key features of its skill from moment to moment, day to day, and year to year. In contrast, for BCI-based skills, sensory feedback is typically unimodal, slow, and discrete; it is generally visual or auditory and may give little information beyond indicating whether the skill is being (or was) performed satisfactorily. Thus, the synthetic heksor lacks the rapid multimodal ongoing guidance it needs for effective adaptation.

**Figure 3.**
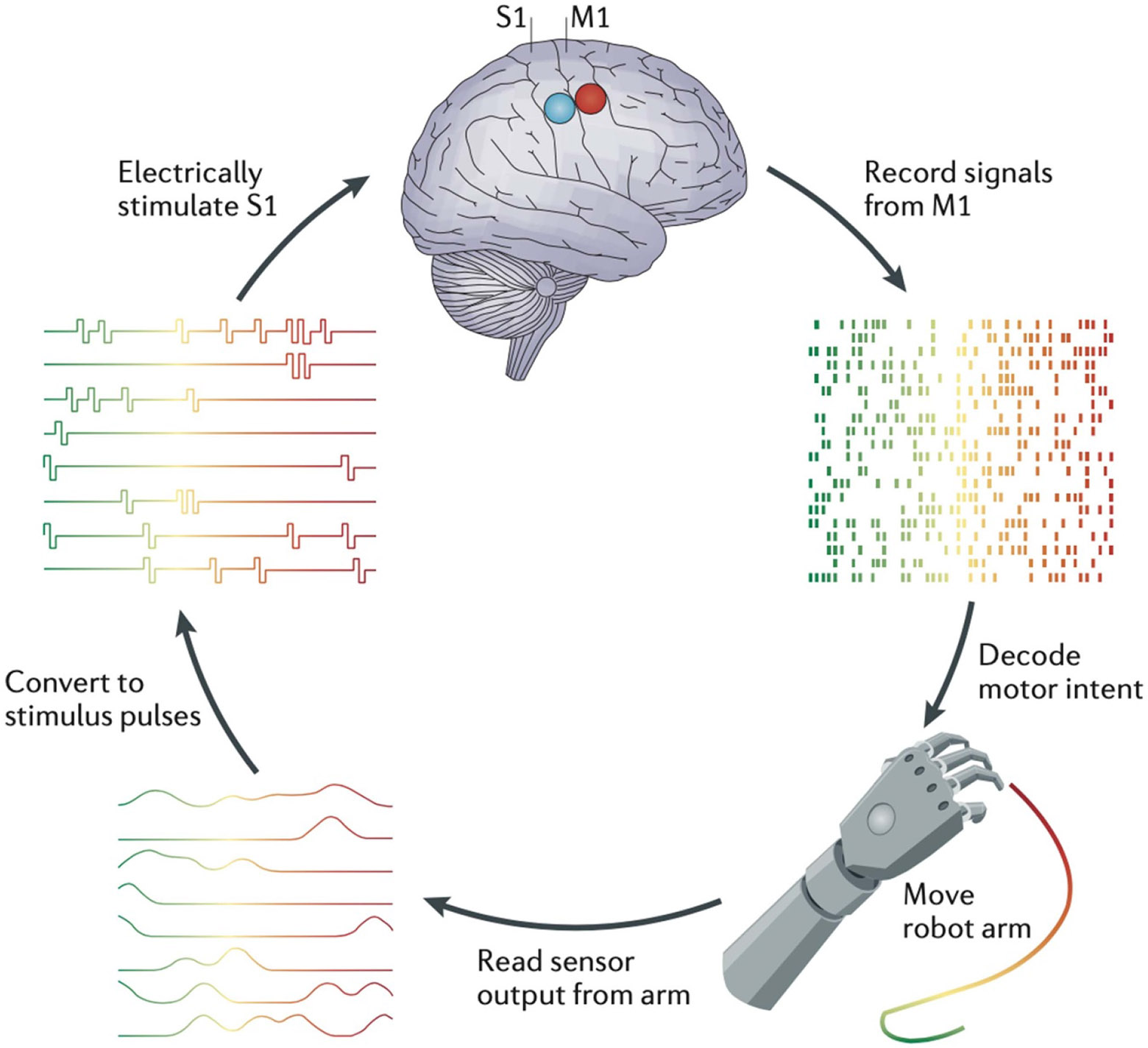
Artificial somatosensory feedback can improve BCI-based prosthesis control. The BCI uses neuronal action potentials from cortical motor areas (e.g. primary motor cortex (M1)) to control movement of a robotic arm. Sensors on the prosthesis send information about its movement and about objects it contacts. This information is translated into electrical stimulation (stimulus pulses) of cortical sensory areas (e.g. primary somatosensory cortex (S1)) by chronically implanted electrode arrays, which is then used to refine the prosthesis movement. (Reproduced from Bensmaia and Miller 2014, with permission from Springer Nature.).

To address this need, studies in animals and humans are exploring the impact of incorporating somatosensory feedback into BCI-based skills (reviewed in Bensmaia and Miller 2014 & Shelchkova et al 2023). As Figure 3 illustrates, at present this sensory feedback during skill performance is usually delivered in the form of precise electrical stimulation of sensorimotor cortex. The stimulation provides the synthetic heksor with rapid, ongoing, and informative sensory feedback akin to that available to natural heksors. In rats, monkeys, and humans, and for a variety of different BCI-based skills, this feedback enhances performance. Furthermore, and perhaps most important, the studies show that the synthetic heksor learns to use this non-natural form of sensory feedback. That is, the CNS part of the synthetic heksor adapts to take advantage of this unusual input. Ideally, this adaptation would be accompanied by concurrent adaptation in the software component of the synthetic heksor that optimizes the effect on performance of the artificial sensory input. For example, in a center-out movement task, the software part of the synthetic heksor might compensate for direction-dependent differences in cortical sensitivity to this sensory input. It could thereby provide the capacity to move with equal ease in any direction. As the incorporation of sensory feedback into BCI-based skills develops, management of co-adaptive interactions between the CNS and software components of the synthetic heksor is likely to be important.

**Figure 4.**
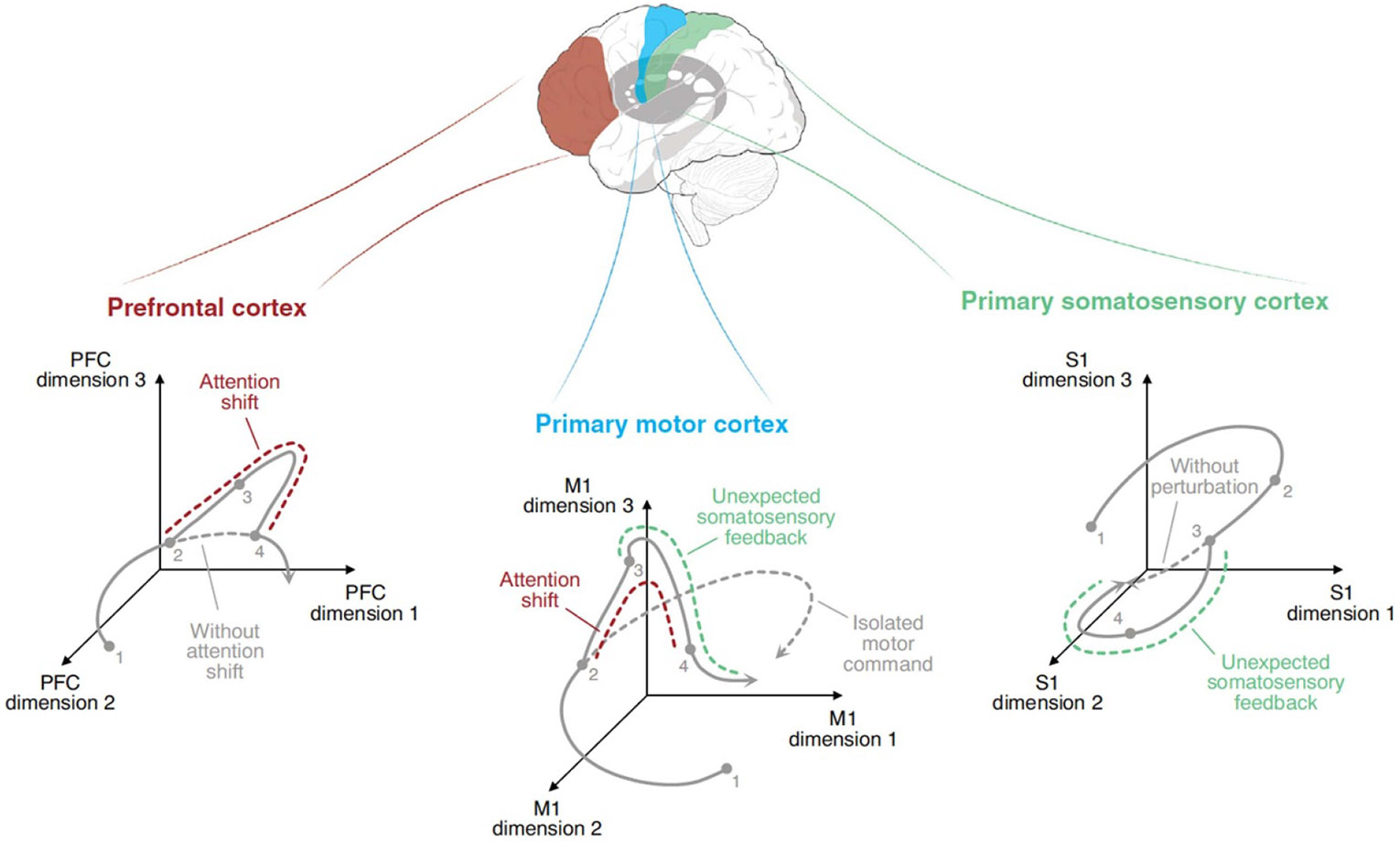
Using signals from multiple areas could improve BCI performance. Neural activity in three major skill-related brain areas: prefrontal cortex (PFC) (left, red); primary motor cortex (M1) (middle, blue); and primary somatosensory cortex (S1) (right, green). Activity in each area during one movement is indicated by its trajectory in a space in which each dimension represents a dominant neural activity pattern. In this example, the BCI user shifts attention during the movement (red dashed line in PFC), and a slight movement perturbation leads to unexpected feedback signals (green dashed line in S1). These attention and feedback signals affect M1 activity (green and red dashed lines) and make it difficult to determine the trajectory of the motor command (gray dashed line). A BCI using only M1 activity may not be able to accurately determine the motor command. Thus, the user may not be able to achieve the intended movement. By using activity recorded from PFC and S1 to subtract the attention and feedback signals from M1 neural activity, a BCI might isolate the trajectory of the motor command in M1, thereby enabling the user to make the intended movement. (Reprinted from Gallego et al, Copyright (2022), with permission from Elsevier.).

The spinal motoneurons that produce muscle-based skills are contacted by descending pathways from many brain regions, as well as by segmental pathways from a variety of peripheral sensory receptors. Thus, a muscle-based skill is the composite product of many different brain regions and peripheral inputs. In contrast, a BCI-based skill is often produced by the activity recorded from only a single brain area, such as primary motor cortex. Studies are now exploring the value of including signals from several cortical regions. Gallego et al (2022) discuss the limitations of using signals from motor cortex alone to control BCI-based movement. While motor cortex activity does reflect intended movement, it also reflects concurrent shifts in attention and concurrent unexpected somatosensory inputs. In the context of BCI-based control based on signals from motor cortex, these other influences are simply noise that impairs BCI recognition of intended movement. As Figure 4 illustrates, a BCI that also records signals from prefrontal and somatosensory cortices might assess concurrent attentional shifts (from prefrontal activity) and unexpected somatosensory activity (from somatosensory activity) and use these assessments to remove non-movement related noise from motor cortex activity, thereby increasing BCI accuracy.

**Figure 5.**
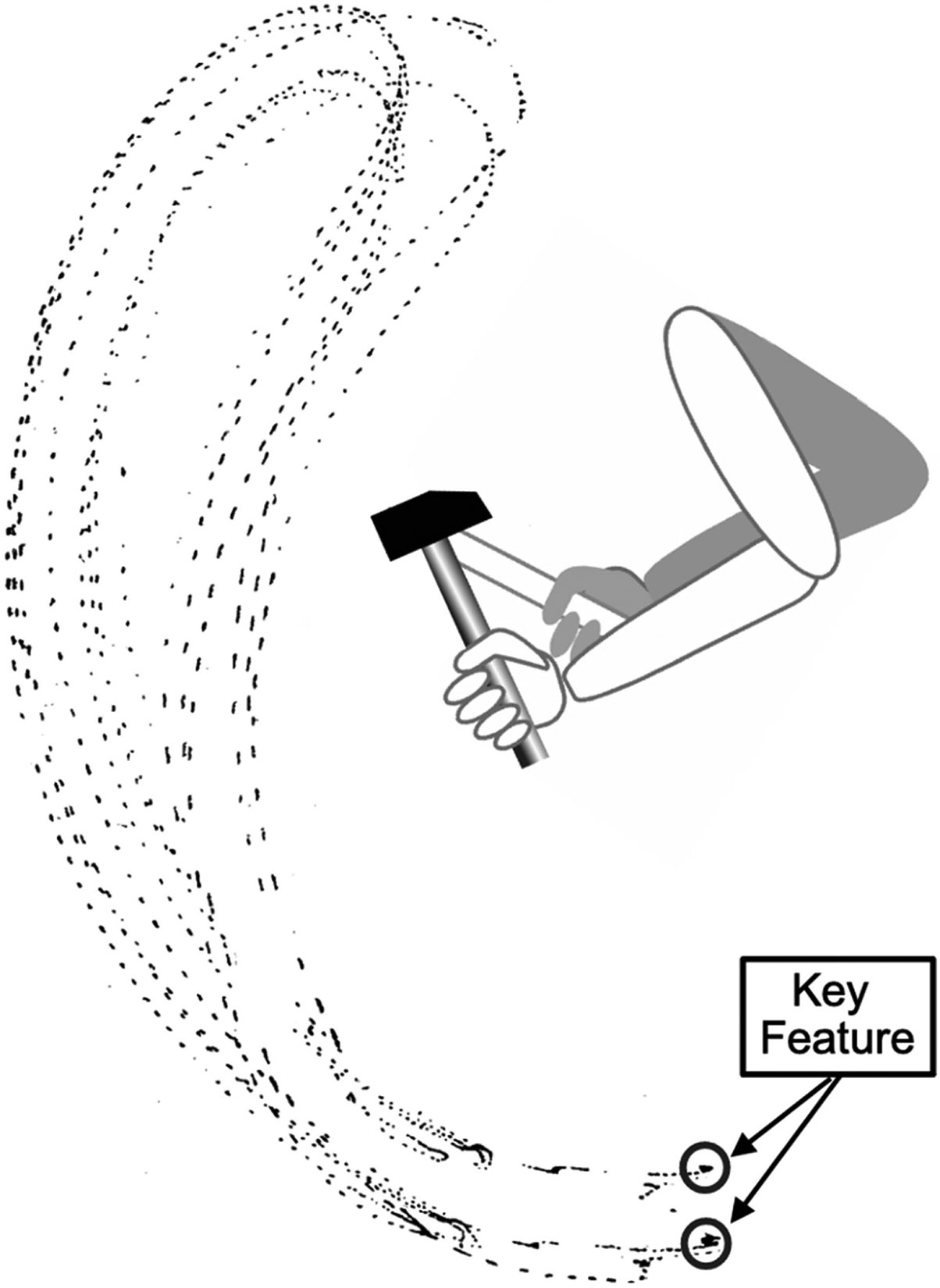
A skill has key features that define it as satisfactory. Almost a century ago, Nikolai Bernstein recorded successive trajectories as a person repeatedly hit the head of a chisel with a hammer. The figure shows multiple hammer trajectories for each of two different chisel locations. The person was very skilled. Nevertheless, the trajectory for a specific chisel location was different every time. The only part of the trajectory that did not change was the point where the hammer hit the chisel; that was the same every time. This was the key feature of the trajectory, the point that determined whether the trajectory was satisfactory or unsatisfactory. The rest of the trajectory could change, as long as the key feature was maintained. (Modified from Bernstein 1967, Sternad 2018).

Another advantage of muscle-based skills that BCI-based skills currently lack is simplicity of output. A muscle-based skill does not require that the timing and level of each muscle’s contribution or the kinematics of movement be exactly the same every time. It requires simply that the key features of the skill—the features that define the skill as satisfactory—are maintained. Figure 5, from Bernstein’s seminal work (1967), illustrates this for the trajectory of movement in a simple action. The only part of the movement trajectory that does not vary from one performance to the next is the point at which the hammer meets the chisel. This is the key feature of the trajectory, the feature that defines the trajectory as satisfactory. In analogous fashion, a BCI controlling body movement at the edge of a cliff might be more reliable if it sought not to control the exact kinematics of movement, but rather to control the key features of body movement, such as posture, balance, right/left symmetry, and center of pressure (especially relative to the cliff edge). To enable this control, the ongoing feedback provided to the synthetic heksor would need to provide ongoing measures that reflect these features.

Many BCI-based skills are abstracted forms of muscle-based skills. Thus, they may require specialized key features. BCI-based movement through an environment might have a key feature that reflects a minimum acceptable distance from an obstruction, and another that reflects a minimum acceptable average velocity toward a desired destination. The recent impressive demonstration of BCI writing based on shapes defined by cortical neural activity (Willett et al 2021) relies on identification of principal components that are essentially key features of this cortical activity.

## 4. Improving synthetic heksor negotiation with natural heksors

Co-adaptation between the CNS and software components of a synthetic heksor is not the only co-adaptive process that affects BCI performance. The CNS component of a synthetic heksor shares neurons and synapses with many natural heksors. Thus, a synthetic heksor is necessarily engaged in the ongoing negotiation (i.e. co-adaptation) through which all the heksors, natural and synthetic, reach a negotiated equilibrium of CNS properties. As discussed above, and illustrated in Figure 2(C), in the present state of BCI development, the result is likely to be less than satisfactory for the synthetic heksor. The many natural heksors and the single synthetic heksor will not adapt to each other so that the synthetic heksor is integrated into a negotiated equilibrium that enables all the heksors, natural or synthetic, to maintain the key features of their skills. Because there is only one synthetic heksor and because it is so different from all the natural heksors, the equilibrium reached is likely to serve the natural heksors while the synthetic heksor loses out. The natural heksors will reliably maintain the key features of their muscle-based skills. The synthetic heksor’s performance will be unreliable; it will be at the mercy of ongoing CNS plasticity that it cannot control and about which it may have no information.

Present possibilities for addressing this problem are limited because little is known about the process through which natural heksors negotiate with each other, beyond the fact that they do. If they did not negotiate, heksors that share neurons and synapses could not reliably maintain the key features of their skills because their skills would be continually disrupted by ongoing adaptations in other heksors. Until the negotiation process is better understood, the measures that could enable a synthetic heksor to negotiate most effectively with natural heksors will not be clear. Nevertheless, several measures likely to encourage and guide this negotiation are discernible. The first has been incorporated into many BCIs.

**Figure 6.**
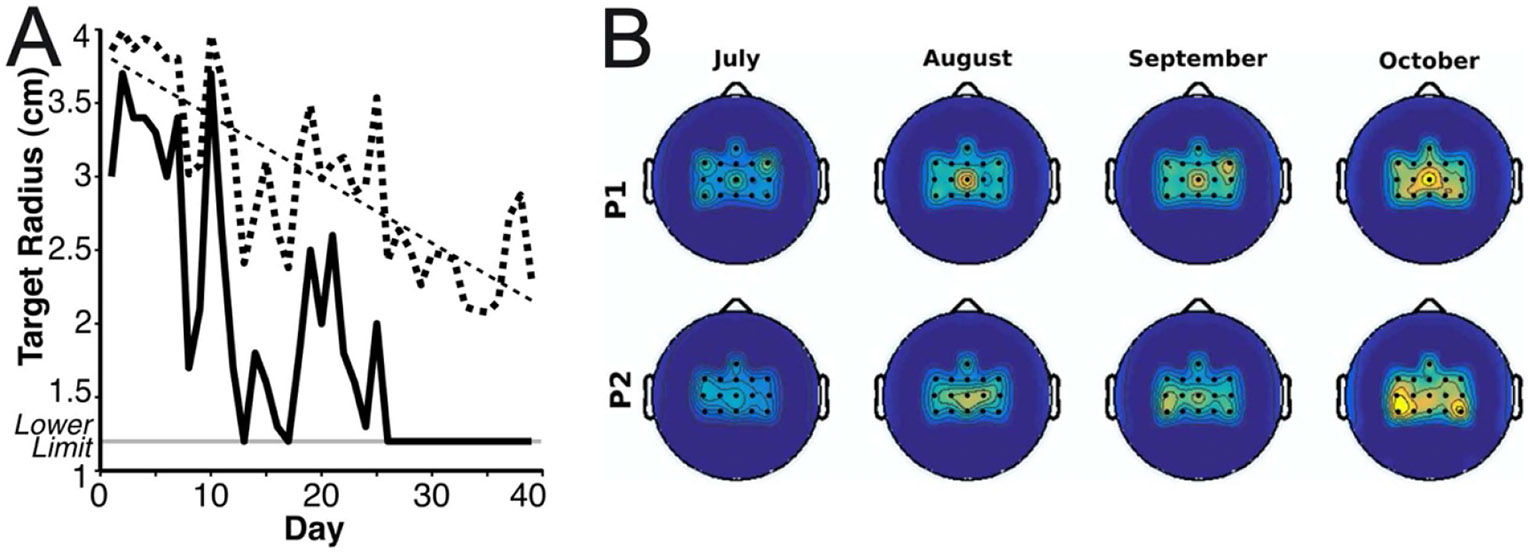
Development of a BCI-based skill may benefit by starting from the brain signals associated with muscle-based performance of the same skill. (A) gradual development of cortical neuron control of three-dimensional cursor movement by a monkey with a microelectrode array implanted in motor cortex. The figure shows daily minimum (solid line) and mean (dotted line) target radii that maintained a 70% target hit rate. The diagonal dotted line is the linear fit of the daily mean (*P <* 0.0001). The bottom horizontal line shows the minimum target radius allowed (1.2 cm). The skill improves as practice proceeds, and the monkey can hit smaller and smaller targets. (From Taylor et al 2002. Reprinted with permission from AAAS.) (B) Progressive development of EEG sensorimotor rhythm control by two people with quadriplegia as they master a BCI-based two-choice game over four months. Topographic maps based on 16 EEG channel locations over sensorimotor cortex show the location and magnitude of control. Brighter color indicates better control. Control at each channel is quantified as the Fisher score of the EEG power spectral density distributions for these two choices in the high *β*-band (22–32 Hz). Each map shows average monthly Fisher scores with inter-channel interpolation. This BCI-based skill improves gradually over months of practice. (Adapted from Perdikis et al 2018. CC BY 4.0.).

A BCI that produces an action, such as reach and grasp with a robotic arm, typically begins by using the brain signals (e.g. neuronal action potentials, EEG rhythms) associated with the comparable muscle-based action (i.e. muscle-based reach and grasp). This choice of starting point—use of the same activity associated with the same action when performed by muscles—may encourage development of a synthetic heksor more compatible with the natural heksors that share the same neurons and synapses. The studies illustrated in Figure 6 used this strategy to achieve control of cursor movement by motor cortex neurons and control of a simple computer game by EEG sensorimotor rhythms (Taylor et al 2002, Perdikis et al 2018). The subsequent progressive development of better BCI performance, illustrated by these examples, provides some support for this strategy.

**Figure 7.**
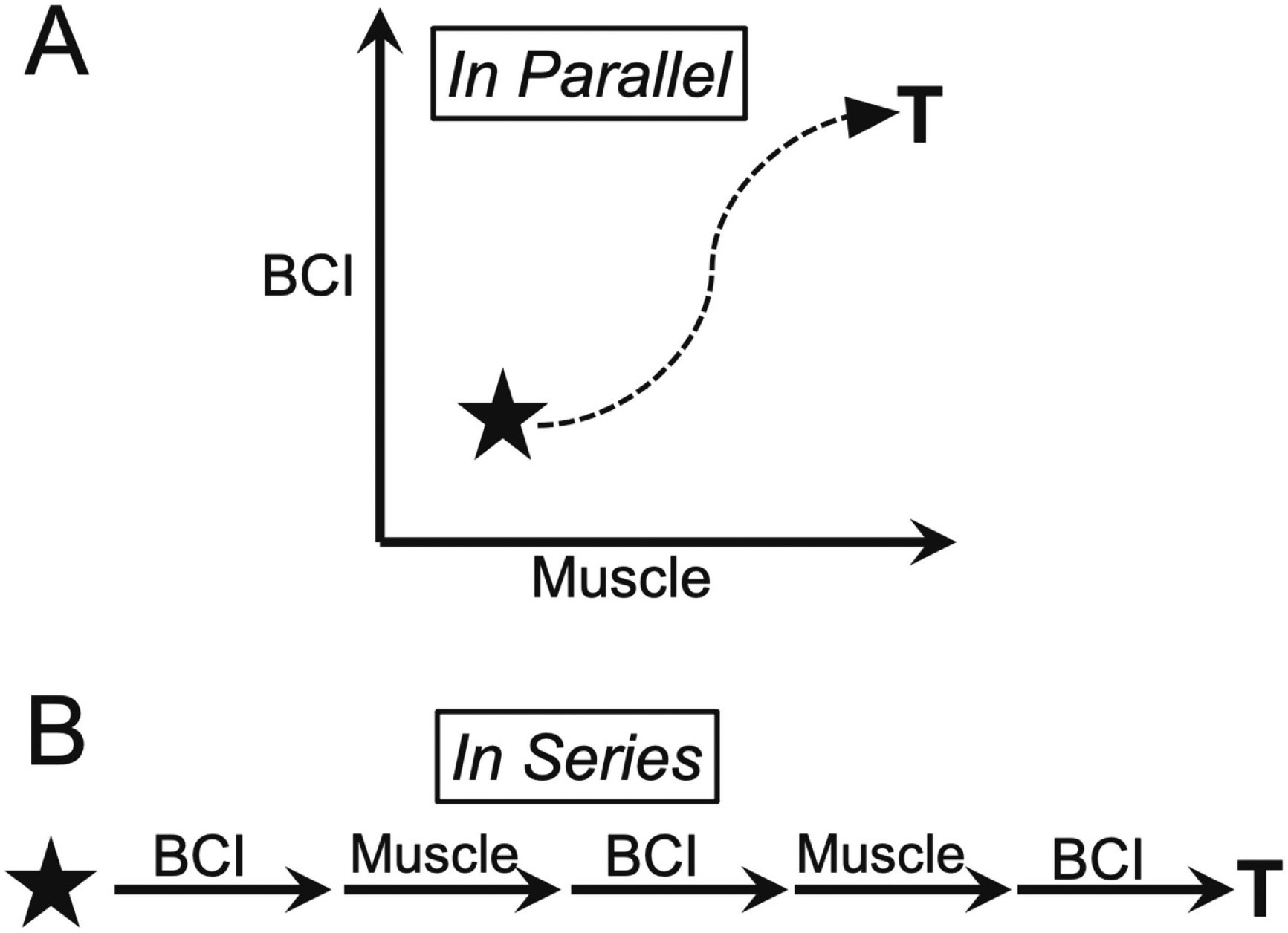
Joint exercises might improve negotiation among natural and synthetic heksors. Synthetic heksor negotiation with natural heksors might be encouraged and guided by a hybrid BCI that requires close cooperation between a synthetic heksor and a natural heksor. (A) Concurrent BCI and muscle control of two-dimensional movement to a target (T). The BCI controls vertical movement; the muscle controls horizontal movement. (B) Alternating BCI and muscle control of one-dimensional movement to a target (T).

Synthetic heksor negotiation with natural heksors might also be encouraged by a hybrid BCI that requires close cooperation between the synthetic heksor and a natural heksor. For example, they might cooperate to control movement to a target. This cooperation might be in parallel or in series (i.e. concurrent or sequential). Figure 7 illustrates these possibilities. And they might share in parallel or in series the steps of a more complex action such as reaching for an object, grasping it, moving it, and releasing it. The BCI might control reaching in one dimension while muscles control it in another; or muscles might control reaching while the BCI controls grasping. With continued development, this approach might evolve into a comprehensive training or exercise regimen for a new synthetic heksor. Its acquisition and initial maintenance would ideally occur in the context of, and be shaped by structured interactions with an appropriate set of natural heksors. The training regimen could be repeated periodically as the person continued to use the BCI. This ongoing structured interaction could help ensure that the synthetic heksor remains well integrated into the negotiated equilibrium maintained by all heksors. In people with minimal remaining muscle control, the synthetic heksor would necessarily interact with the few natural heksors that remained functional. In this reduced situation, the negotiation between the natural heksors and the synthetic heksor might be simpler and more balanced, and the result more satisfactory for the synthetic heksor as well as for the natural heksors.

## 5. Conclusion

If BCIs are to attain the reliability needed for actions in which error probability must be zero or very close to zero, neural engineering research that improves the recording and analysis of brain signals must be coupled with neuroscience research that enables BCI-based skills to emulate natural muscle-based skills. A BCI-based skill is produced by a synthetic heksor, a network of neurons, synapses, and software that produces the skill and changes as needed to maintain the skill’s key features. Like natural heksors, a synthetic heksor benefits from multimodal, rapid, and ongoing sensory feedback, from using signals from more than one brain area, and from maintaining the key features of the skill rather than every detail of its performance. A synthetic heksor also requires effective co-adaptation between its CNS and software components. And, because a synthetic heksor shares neurons and synapses with natural heksors, it needs effective integration into the negotiated equilibrium of CNS properties that enables each heksor, natural or synthetic, to maintain its key features. Thus, a synthetic heksor could benefit from training protocols that require close cooperation with natural heksors. With appropriate combination of neural engineering and neuroscience research, BCI-based skills could eventually become as reliable as muscle-based skills.

## Data Availability

No new data were created or analysed in this study.
